# The Great Masquerader: Syphilis Mimicking Papilledema and Traction Alopecia

**DOI:** 10.7759/cureus.7391

**Published:** 2020-03-24

**Authors:** Michelle A McNally, Angela Murira, Christen M Dillard, Gabriel Aisenberg

**Affiliations:** 1 Dermatology, John P. and Kathrine G. McGovern School of Medicine, University of Texas Health Center at Houston, Houston, USA; 2 Internal Medicine, John P. and Kathrine G. McGovern Medical School, University of Texas Health Science Center at Houston, Houston, USA

**Keywords:** syphilis, syphilitic alopecia, papillitis, papilledema, traction alopecia

## Abstract

Syphilis is often referred to as “the great masquerader,” as it may present with a wide array of clinical symptoms and may mimic a variety of other diseases making diagnosis challenging. We report the case of a young, married woman who presented with a one-month history of significant hair loss, unintentional weight loss, blurred vision, and intermittent headaches. In addition, she endorsed positional dizziness and intermittent arthralgias. Physical exam was only remarkable for a non-scarring alopecia over the frontal marginal hairline and optic disc edema on fundoscopic exam. Laboratory tests were largely unremarkable except for a rapid plasma reagin titer of 1:128 and a positive confirmatory treponemal test. Cerebrospinal fluid analysis showed lymphocytic pleocytosis and negative Venereal Disease Research Laboratory test. Opening pressure was 15 cm H_2_O twice, ruling out papilledema. She was treated with 4 million units of intravenous penicillin every four hours for 14 days, and her symptoms improved. A diagnosis of syphilis should remain high on the differential diagnosis in patients with unexplained hair loss or ocular abnormalities.

## Introduction

Syphilis is often referred to as “the great masquerader,” as it may present with a wide array of clinical symptoms and may mimic a variety of other diseases making diagnosis challenging. The presentation depends on the disease stage: hair loss is commonly described in secondary syphilis, while varied ocular involvements have been described in secondary and tertiary syphilis [1,2]. Papilledema, a swelling of the optic discs, presents when the intracranial pressure is above 25 cm H_2_O; similar fundoscopic findings, however, can be seen with neuritis and perineuritis of the ocular nerve, as seen in some syphilitic patients [3,4].

We report a case of syphilis in a young, married woman who presented with hair loss, unintentional weight loss, headaches, and blurred vision in the presence of optic disc edema.

## Case presentation

A 30-year-old married Hispanic female with no past medical history presented with a one-month history of significant hair loss, black and gray floaters in her left eye, blurred vision, headaches, and an unintentional 23-pound weight loss. Her headaches were intermittent in nature and located in the bilateral occipital and frontal regions. They were not associated with auras, photophobia, lacrimation, positional changes, or changes in vision. She reported occasional nausea and non-bloody emesis in association with her headaches. Her weight loss occurred without changes in her diet or appetite. She also noted positional dizziness when bending over and intermittent arthralgias in her knee joints. Her symptoms began abruptly in a one-month period. She denied having a prior history of similar symptoms.

**Figure 1:**
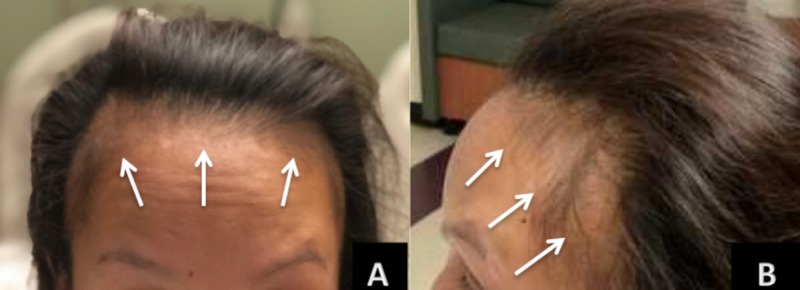
Non-scarring frontal alopecia (A) Non-scarring alopecia is present on the frontal marginal region (arrows) of the scalp. (B) Marginal alopecia involving the frontal temporoparietal region (arrows) of the scalp.

**Figure 2:**
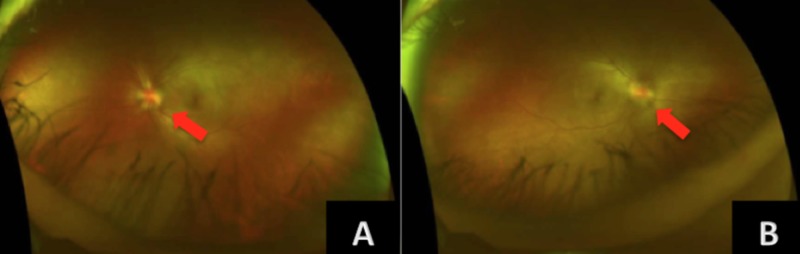
Bilateral disc edema (A) Left optic disc edema, blurred optic head margins 360 degrees (arrows), elevated optic nerve 360 degrees, no peripapillary hemorrhage or vessel obstructions. (B) Right eye shows similar findings.

On physical examination, non-scarring alopecia was present over the frontal marginal hairline (Figure 1A, 1B). Her eyebrows, skin, and mucous membranes appeared normal. Fundoscopic examination revealed bilateral optic disc edema (Figure 2A, 2B), classified as grade III, which is characterized by a peripapillary circumferential halo with decreased translucency and at least one obscured major vessel as it passes the optic disc margin [5]. The remainder of the physical exam was unremarkable.

The results of laboratory investigations including complete blood count, blood chemistry, thyroid function, cerebrospinal fluid analysis (CSF), and immunologic labs were within normal limits other than a microcytic anemia (hemoglobin of 10.1 g/dL) and an elevated erythrocyte sedimentation rate of 50 mm/hr.

Initial CT of the head showed a partially empty sella with no other acute abnormalities. Follow-up MRI of the orbit and head showed flattening of the bilateral globes consistent with papilledema and intracranial hypertension. No enhancement of the optic nerve sheath was present. A lumbar puncture (LP) measured a normal opening pressure of 15 cm H_2_O twice, thus ruling out a diagnosis of papilledema. CSF analysis showed mild lymphocytic pleocytosis (16 white blood cells/μL). CSF Venereal Disease Research Laboratory test was negative.

Further laboratory testing revealed a serum rapid plasma reagin (RPR) titer of 1:128, and a confirmatory test for antibodies to Treponema pallidum using fluorescent treponemal antibody absorption was positive, confirming the diagnosis of syphilis. Subsequent testing for antibodies to the HIV was negative. The patient was started on intravenous (IV) penicillin G (4 million units every four hours for 14 days) for the treatment of secondary syphilis with ocular involvement. Over the following days, the patient reported improvement in the floaters in her left eye, resolution of the blurry vision, headaches, positional dizziness, and arthralgias. She gained five pounds from admission to discharge. She is scheduled to return for a repeat RPR and follow-up ophthalmologic and neurologic examination three months after having completed therapy. 

## Discussion

Syphilis has seen a drastic resurgence in incidence in recent years. Primary and secondary syphilis have increased by 71% since 2014. The majority of new cases are attributed to men who have sex with men, though rates in women have also seen a significant rise in the past five years. This is of particular concern as the increase of syphilis in women coincides with a substantial concomitant increase in rates of congenital syphilis [6].

Syphilis is a sexually transmitted systemic disease caused by the gram-negative spirochete, Treponema pallidum. Clinically, the progression of syphilis is subdivided into four stages: primary, secondary, latent, and tertiary. Primary syphilis is characterized by a painless genital ulcer, which resolves spontaneously after several weeks. Secondary syphilis typically presents one to two months later with generalized systemic symptoms, such as fever, malaise, headaches, weight loss, and arthralgias [4,7]. A disseminated mucocutaneous macular or papular rash is the most common physical manifestation of secondary syphilis and frequently involves the palms and soles [8]. Alopecia also presents during the secondary stage [4]. Latent syphilis presents with positive serologic findings in the absence of clinical symptoms and may last for years [8]. Tertiary syphilis occurs years later in a subset of untreated patients and presents as cardiovascular syphilis, neurologic syphilis, or gummatous syphilis [9].

Syphilitic alopecia is uncommon, occurring in 4% to 11% of secondary syphilis cases and is characterized by discrete patches of non-scarring alopecia with a classic “moth-eaten” appearance [9,10]. In some cases, syphilitic alopecia may be the only presenting feature of secondary syphilis on physical examination [10]. Even less common, a diffuse pattern hair loss, termed essential syphilitic alopecia, may present in latent syphilis in the absence of other cutaneous findings [11]. Penicillin is an effective treatment for syphilitic alopecia in most cases.

Traction alopecia is a trauma-induced non-scarring alopecia caused by chronic, excessive traction of the hair shaft from pulling hairstyles [12]. It presents on the temporoparietal frontal margins of the hairline. African-American women are more frequently affected. Traction alopecia is reversible in early stages, but may later develop into scarring alopecia that is irreversible [12]. Although the pattern of hair loss seen is our patient is similar in presentation to traction alopecia, our patient’s abrupt onset, severe hair loss in the absence of any changes to hairstyle is more likely explained by syphilitic alopecia.

Ocular syphilis may present during any stage of syphilis and may affect virtually any eye structure; however, panuveitis and posterior uveitis are the most common complications [4,13]. Cases of ocular syphilis are predominantly reported in HIV-positive patients [13]. Patients with ocular syphilis may present with complaints of blurred vision, floaters, eye pain, photophobia, or vision loss [2]. Patients with syphilis should be assessed for ocular abnormalities and those with ocular syphilis should undergo LP with CSF evaluation [13].

Papillitis, optic neuritis, and optic perineuritis are all terms previously used to describe syphilitic involvement of the optic nerve; however, Chen et al. recently proposed the term incipient syphilitic papillitis to describe optic nerve edema in syphilis patients with preserved visual acuity, normal intracranial pressure, and a lack of optic nerve sheath enhancement on MRI [14].

With prompt diagnosis and treatment, the prognosis for ocular syphilis is favorable. Syphilis patients with ocular complaints should undergo immediate ophthalmologic evaluation, as delay in treatment may lead to blindness [2,13]. Patients should be managed collaboratively with an ophthalmologist [6]. The treatment of ocular syphilis is the same as for neurosyphilis (even with normal CSF findings), that is a 10- to 14-day course of 3 to 4 million units of IV aqueous penicillin G given every four hours, or a continuous IV infusion of 24 units of insulin daily for 10-14 days [4]. If compliance is a concern, an alternative 10- to 14-day regimen of 2.4 million units of intramuscular procaine penicillin G, once daily, plus 500 mg of oral probenecid, four times a day, may be considered [6]. 

Repeat CSF examination should be performed in patients who present with abnormal CSF findings [6]. Clinical and serologic follow-up with repeat titers should occur at six and twelve months post-treatment, although more frequent evaluation may be necessary if there is concern for reinfection or loss to follow-up [6].

## Conclusions

The spectrum of presentation of syphilis remains broad and vague and includes alopecia and eye involvement. In settings like our patient’s, papilledema should be confirmed or ruled out by demonstrating a consistent elevated opening pressure. When this is not the case, syphilitic papillitis is a likely explanation of the ocular finding. A prompt treatment can potentially reverse both ocular syphilis and syphilitic alopecia.
